# Spinal Cord Cocainization

**Published:** 1900-11

**Authors:** 


### EDITORIAL NOTES AND COMMENTS.

The Business office of THE JOURNAL-RECORD is No. 64 Marietta Street.

The Editorial office is 318-319-320 The Prudential.

Address all Business communications to Dr. M. B. Hutchins, Mgr.

Make remittances payable to THE ATLANTA JOURNAL-RECORD OF MEDICINE.

On matters pertaining to the Editorial and Original Communications address Dr. Bernard Wolff, Atlanta.

Reprints of original articles will be furnished at cost price. Requests for the same should always be made *on the manuscript*.

We will present, post-paid, on request, to each contributor of an original article, twenty (20) marked copies of THE JOURNAL-RECORD containing such article.

### SPINAL CORD COCAINIZATION.

THE increasing fear of an anesthetic among the profession as adding an element of danger to surgical procedures, not in themselves dangerous, has paved the way for ready acceptance of any method that promises analgesia with diminished hazard. Medullary cocainization, though not yet thoroughly tested, holds out strong hope of accomplishing this purpose. The suggestion of this amplification of the use of the anesthetic effects of cocain is due to J. Leonard Corning of New York. With him, however, the theory never went as far as practical demonstration. Quincke's lumbar puncture for the purpose of obtaining cerebrospinal fluid for diagnostic purposes supplied another pedestal to the theory. It remained for Bier, of Kiel, to conjoin these elements and carry out the observations in practice. Tuffier, of Paris, took up the work of Bier with Gallic enthusiasm and gave the impulse to the method which has been felt all over the world. American surgeons, quick to follow the initiative, have tested Tuffier's method, and already gratifying successes have been reported by J. B. Murphy, S. Marx (in obstetrics), J. R. Goffe, and R. Matas. The last gives an exhaustive review of the subject in a paper read before the Louisiana Medical Society.

Tuffier's technic, that which is generally adopted, is as follows: The patient sits upright and is told to lean forward. In this way there is occasioned a separation between the lumbar vertebræ. The space between the 4th and 5th lumbar vertebræ is that chosen for the injection, and the seat is marked by the index-finger of the left hand. The hypodermic-needle is than taken in the right hand and the point inserted a fourth of an inch to the right of the middle line at the point of the indicating finger. The needle is then thrust in until it reaches the subdural space, which fact will be announced by the escape of cerebro-spinal fluid. The barrel of the instrument containing a freshly prepared, carefully sterilized two per cent. solution is attached and the fluid slowly injected. At the end of a short time, eight to ten minutes, anesthesia is produced, beginning in the region below the seat of the injection and slowly ascending to the axillary line.

More than a thousand operations have up to the present been performed, including laparotomies, herniotomies, amputations of the extremities, and almost without exception painlessly. There has been no fatality and no accident of moment. The patients have complained of faintness and nausea, nothing further. The method has a wide field of application, and it is most devoutly to be wished that future developments will not mar its brilliant promise.

